# Characterization of the Invasive, Multidrug Resistant Non-typhoidal *Salmonella* Strain D23580 in a Murine Model of Infection

**DOI:** 10.1371/journal.pntd.0003839

**Published:** 2015-06-19

**Authors:** Jiseon Yang, Jennifer Barrila, Kenneth L. Roland, Jacquelyn Kilbourne, C. Mark Ott, Rebecca J. Forsyth, Cheryl A. Nickerson

**Affiliations:** 1 Center for Infectious Diseases and Vaccinology, The Biodesign Institute, Arizona State University, Tempe, Arizona, United States of America; 2 Biomedical Research and Environmental Sciences Division, NASA Johnson Space Center, Houston, Texas, United States of America; 3 School of Life Sciences, Arizona State University, Tempe, Arizona, United States of America; Oxford University Clinical Research Unit, VIET NAM

## Abstract

A distinct pathovar of *Salmonella enterica* serovar Typhimurium, ST313, has emerged in sub-Saharan Africa as a major cause of fatal bacteremia in young children and HIV-infected adults. D23580, a multidrug resistant clinical isolate of ST313, was previously shown to have undergone genome reduction in a manner that resembles that of the more human-restricted pathogen, *Salmonella enterica* serovar Typhi. It has since been shown through tissue distribution studies that D23580 is able to establish an invasive infection in chickens. However, it remains unclear whether ST313 can cause lethal disease in a non-human host following a natural course of infection. Herein we report that D23580 causes lethal and invasive disease in a murine model of infection following peroral challenge. The LD_50_ of D23580 in female BALB/c mice was 4.7 x 10^5^ CFU. Tissue distribution studies performed 3 and 5 days post-infection confirmed that D23580 was able to more rapidly colonize the spleen, mesenteric lymph nodes and gall bladder in mice when compared to the well-characterized *S*. Typhimurium strain SL1344. D23580 exhibited enhanced resistance to acid stress relative to SL1344, which may lend towards increased capability to survive passage through the gastrointestinal tract as well as during its intracellular lifecycle. Interestingly, D23580 also displayed higher swimming motility relative to SL1344, *S*. Typhi strain Ty2, and the ST313 strain A130. Biochemical tests revealed that D23580 shares many similar metabolic features with SL1344, with several notable differences in the Voges-Proskauer and catalase tests, as well alterations in melibiose, and inositol utilization. These results represent the first full duration infection study using an ST313 strain following the entire natural course of disease progression, and serve as a benchmark for ongoing and future studies into the pathogenesis of D23580.

## Author Summary

A deadly form of non-typhoidal *Salmonella* has emerged as a major cause of invasive disease in sub-Saharan Africa. Initial genomic profiling of this novel *Salmonella* sequence type, ST313, indicated that although it is technically classified as *S*. Typhimurium (a serovar characterized by a broad host range), it may be evolving towards becoming a more human-specific, ‘typhoid-like’ pathogen. However, it was recently demonstrated that ST313 strains were indeed able to establish an invasive and damaging infection in chickens. Despite these important findings, it remains unclear whether ST313 is able to cause lethal disease in a non-human host, since no study has yet followed the entire natural course of disease progression. As such, there are no data available concerning the median lethal dose (LD_50_) of any ST313 strain. This is an important metric, as the LD_50_ value will serve as a benchmark for mechanistic studies focused on understanding the relationship between virulence and the phenotypic and molecular genetic attributes associated with ST313 infections. Here we report that D23580 causes lethal disease in BALB/c mice and determined the LD_50_ following peroral challenge. Phenotypic characterization revealed distinct differences in tissue distribution, acid stress resistance, and biochemical utilization between D23580 and the ‘classic’ Typhimurium strain SL1344.

## Introduction

Infectious diseases caused by multidrug resistant (MDR) pathogens continue to be a major global health crisis and challenge current treatment regimens. Invasive non-typhoidal salmonellae (iNTS) are a leading cause of bloodstream infections in sub-Saharan Africa, and are of serious concern due to high rates of morbidity and mortality coupled with increasing problems of MDR [1–4]. iNTS have replaced pneumococcus as the most frequent cause of bacteremia in several countries, with *Salmonella enterica* serovar Typhimurium (*S*. Typhimurium) identified as one of the most common serovars recovered from patients with iNTS infections [1–7]. There is currently no vaccine available for prevention of iNTS disease in humans.

Kingsley et al. first reported in 2009 that a phylogenetically distinct pathovar of *S*. Typhimurium belonging to a novel multilocus sequence type (MLST) designated as ST313 had emerged as a significant cause of morbidity and mortality among HIV-positive adults and children suffering from malaria, severe anemia and/or malnutrition [1]. Case fatality rates are high, ranging from 20–25% in children and extend up to ~50% in HIV-infected adults [1]. Recurrence of the disease due to recrudescence of the same strain and/or reinfection with a separate iNTS strain occurs frequently, and can lead to high mortality rates over the long term [8]. No animal reservoir has been identified thus far for ST313, and it has been suggested that unlike other ‘classical’ foodborne NTS infections, which are often transmitted via zoonotic routes, ST313 strains may pass primarily through human-to-human contact, [5,6]. Rapid and accurate diagnosis is often hindered by the non-specific clinical symptoms associated with the disease, which most commonly present only as a fever with a subset of patients experiencing splenomegaly [1,3,9]. There is also a marked lack of gastroenteritis in most cases that is often characteristic of NTS infections (<50% of cases) [1,3]. Moreover, the increasing problem of MDR to commonly used antibiotics including ampicillin, trimethoprim-sulfamethoxazole, and chloramphenicol, presents additional challenges in these impoverished regions, as cost and availability can preclude the use of alternative antimicrobial agents [2,8]. Due to the lack of blood diagnostic facilities in many regions where ST313 infections are the most rampant, treatment using an inadequate antibiotic regimen following a misdiagnosis often fails to combat the infection [3]. However, even with the appropriate diagnosis and implementation of a rigorous antibiotic regimen, the average case fatality rates still hover around 25% [2].

There is urgent need to understand the pathogenic strategies used by these deadly iNTS strains to cause disease in order to facilitate the development of novel diagnostic tools and for the design of effective treatments and prevention strategies. In recent years, attention has been given towards understanding the distinctive cellular and humoral immune responses associated with ST313 infections [3,10–17] as well as the unique genotypic and phenotypic characteristics associated with the ST313 pathovar [1,5,6,14,18–25]. Kingsley et al. performed MLST profiling of 51 iNTS isolates recovered from Malawi and Kenya during the peak of the Blantyr epidemic and identified ST313 as the dominant genotype responsible for iNTS disease in the region [1]. Whole genome sequencing of D23580, a representative MDR ST313 clinical isolate from a pediatric patient in Malawi, indicated that the strain had undergone genome reduction similar to that of other human-restricted serovars like *Salmonella enterica* serovar Typhi (S. Typhi) [1]. D23580 was found to contain a novel prophage repertoire, as well as the presence of a large insertion of MDR genes on the large pSLT-BT plasmid. Importantly, a large number of the pseudogenes and deletions identified are consistent with what has been observed for *S*. Typhi [1]. These findings, together with the previous clinical and epidemiological observations, including a routine lack of gastroenteritis and evidence of human-to-human transmission, suggested the possibility that the ST313 pathovar may be evolving towards a more host-restricted phenotype similar to that of *S*. Typhi [1,18].

Recent studies have confirmed that despite some similarities to *S*. Typhi, D23580 still retains a broad host tropism characteristic of *S*. Typhimurium ([14,18], this work). Tissue distribution studies conducted in chickens by Parsons et al. were the first to demonstrate that D23580 is able to infect chickens and displays an invasive phenotype [18]. D23580 colonized the ceca, spleen and liver, and elicited a rapid inflammatory CXC chemokine response in the intestine [18]. Comparisons made to ST19 isolates 4/74 and F98 indicated that D23580 invaded deeper into the spleen and liver, and colonized the intestinal tract at lower levels. Subsequent studies conducted by Herrero-Fresno et al. in C57/BL6 mice sought to understand the role of the uncharacterized gene, *st313-td*, in ST313 pathogenesis [14,20]. Competition experiments between the wild type ST313 strain 02-03/002 and the *st313-td* deletion mutant following intraperitoneal (i.p.) challenge indicated that while both the wild type and the mutant were able to colonize the spleen, deletion of *st313-td* led to a severe decrease in invasiveness. These findings correlated well with human clinical data, wherein the presence of the *st313-td* gene in S. Typhimurium correlated strongly with invasiveness with respect to systemic infection [14]. Moreover, it was found that while the presence of the gene did not impact invasion into Int-407 intestinal epithelial monolayer cultures, it did affect survival in J774 macrophages [14]. Along these lines, it was recently found that ST313 strains were phagocytosed more efficiently and were highly resistant to killing by macrophages of both human and mouse origin, relative to ST19 isolates [26].

While the previous studies have demonstrated that ST313 strains are capable of causing a systemic infection in both chickens and mice, to our knowledge no study to date has yet assessed the lethality of ST313 in animals. Herein we the report the median lethal dose (LD_50_) of the ST313 strain D23580 in 8-week-old female BALB/c mice following peroral (p.o.) infection. Comparisons made between the tissue colonization patterns of D23580 and the classic *S*. Typhimurium strain SL1344 revealed distinct differences between the two strains and indicated that D23580 was able to more rapidly colonize the spleen, mesenteric lymph nodes and gall bladder in mice. In addition, several assays including acid stress, motility and biochemical profiling were conducted in order to better understand how these factors could play a role in the pathogenesis of D23580.

## Methods

### Ethics statement

This study was reviewed and approved by the Arizona State University Institutional Animal Care and Use Committee (IACUC) under protocol number 14-1343-R. All animals were housed in accordance with the American Association for Laboratory Animal Care (AALAC) standards, provided unlimited access to food and water, and handled in accordance with the Animal Welfare Act and Institutional Animal Care and Use Committee (IACUC) regulations. Experiments involving animals were conducted in a facility fully accredited by the Association for Assessment and Accreditation of Laboratory Animal Care International (Unit #000765) and an assurance is on file with the Office for Laboratory Animal Welfare (#A3217-01). Experiments were planned and conducted utilizing the three R's (reduce, replace and refine), which included environmental enrichment, veterinary oversight, numbers reflecting statistical significance and the use of appropriate analgesics and anesthesia when appropriate. Mice used in this study were euthanized by CO_2_ asphyxiation, which is consistent with the most recent recommendations of the American Veterinary Medical Association (AVMA) Panel on Euthanasia. Cervical dislocation or secondary thoracotomy was used as a subsequent secondary measure.

### Bacterial strains and growth conditions

Bacterial strains used in this study are listed in Table 1 [1,27–29]. For all animal studies and stress assays, bacterial cultures were initiated in Lennox broth (LB) with aeration (180 rpm) overnight for 15 hours at 37°C. The following day, overnight cultures were inoculated into 50 mL sterile LB at a 1:200 dilution and subsequently grown to late log/early stationary phase at 37°C with aeration. To confirm that all bacterial strains used in animal studies and stress assays were at the same phase of growth for all studies, growth curves were performed for those strains under these conditions by plating on LB agar for viable colony-forming units (CFU) and measuring the corresponding optical density at 600 nm (OD_600_) (S1 Fig).

**Table 1:** Bacterial strains.

Genus and subspecies	Strain	Characteristics	References
*S*. Typhimurium	SL1344	Wild-type, (Sm^R^)	(27)
*S*. Typhimurium	D23580	ST313, clinical isolate, (SmSuChAW^R^, Km^S^)	(1)
*S*. Typhimurium	A130	ST313, clinical isolate, (SuKmAW^R^, SmCh^S^)	(1)
*S*. Typhi	Ty2	Wild-type, RpoS^-^,Cys^-^,OD_1_:H_d_:-:Vi, V form	(29)

Sm, streptomycin; Su, sulphonamide; Ch, chloramphenicol; Km, kanamycin; A, ampicillin; W, trimethoprim

^R^, resistant

^S^, sensitive.

### Virulence studies

**Fig 1:**
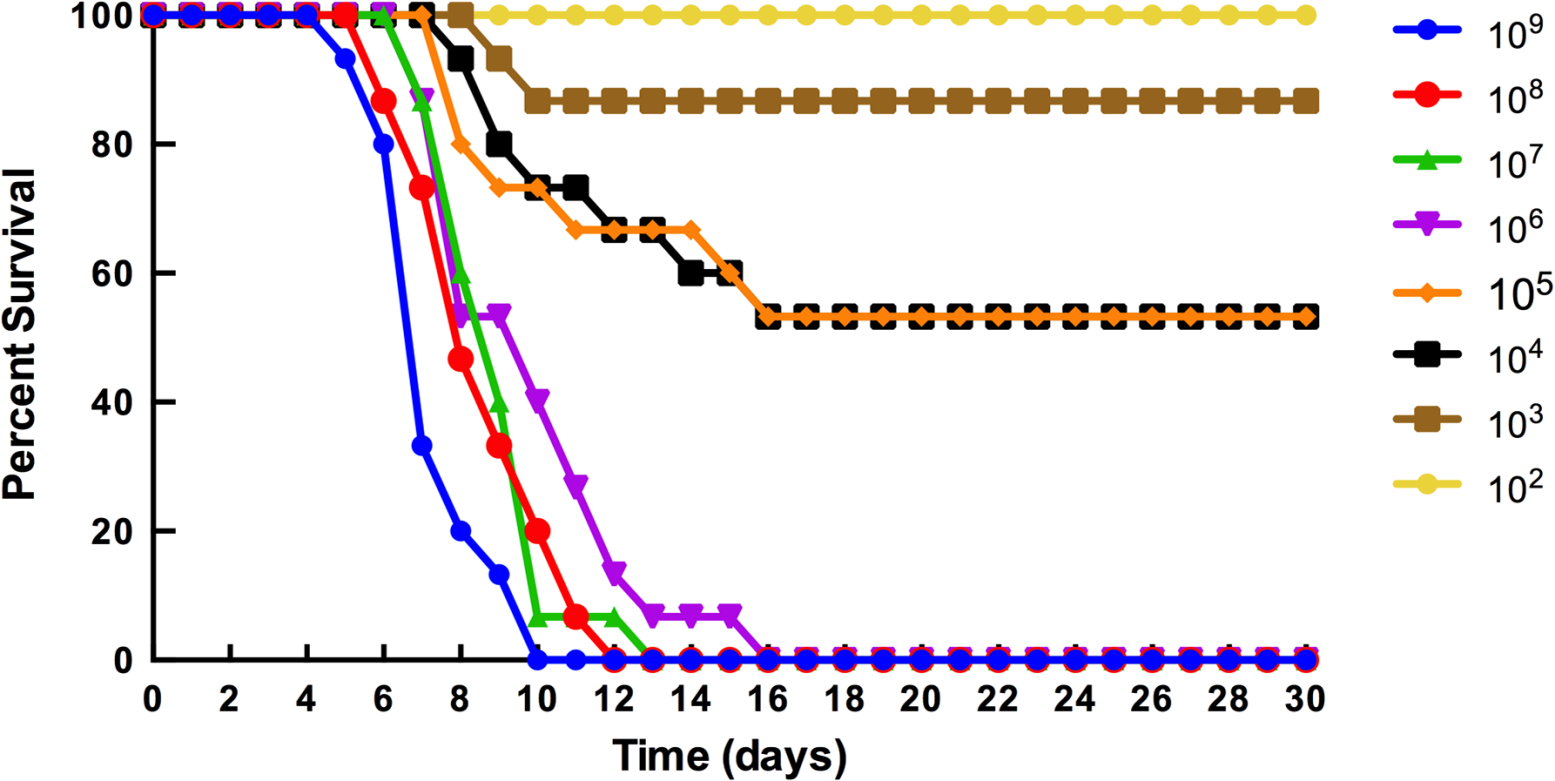
Survival of mice following peroral infection with D23580. D23580 was cultured to late log phase and administered p.o. to 8-week-old female BALB/c mice at inoculum titers ranging from 10^2^–10^9^ CFU per dose. The data shown represent the combined results from three independent trials. The median lethal dose was determined by the method of Reed and Muench [31] using two of these independent trials (see Methods section). The percent survival is defined as the percentage of mice surviving at the indicated number of days post-infection.

The virulence of D23580 in 8-week old female BALB/c mice (Charles River Laboratories) was determined by p.o. administration using standard protocols described previously [30]. D23580 was cultured to late log/early stationary phase as described above and harvested by centrifugation at 7,000 rpm for 10 minutes. Pellets were resuspended in 1 mL of buffered saline containing 0.01% gelatin (BSG) to a dose of approximately 1x10^9^ CFU per 20 μl. A series of 10-fold dilutions was performed in BSG down to 1x10^2^ CFU per 20 μl dose. Animal inoculations for the determination of the 50% lethal dose (LD_50_) values were performed as described previously [30]. Studies were performed in biological triplicate with five mice per dose. The LD_50_ value was calculated using two of these independent trials, since in the initial trial the dosage was not yet optimized and there was no group with 100% survival, which is a requirement to calculate the median lethal dose using the method of Reed and Muench [31]. Time-to-death (Fig 1) was plotted to include the results from all three trials. Mice were monitored for up to 30 days for both the LD_50_ and time-to-death studies.

### Tissue distribution studies

Dissemination of D23580 and SL1344 in mice was assessed by separate p.o. inoculations into 8-week old female BALB/c mice. Bacteria were cultured and harvested as described above. Approximately 5x10^8^ CFU per 20 μl was used for inoculating each mouse. Three groups of five mice were infected with D23580, and a matching number of groups with SL1344. Quantitation of viable bacteria in tissues and organs at days 1, 3 and 5 post-infection was performed as described previously [30]. Briefly, mice were euthanized with CO_2_, and tissues of interest were promptly dissected and weighed. Bacteria were enumerated from the following tissues/regions: Peyer’s patches (7–11 per mouse), intestinal contents, intestinal wall (small and large intestines with Peyer’s patches removed), mesenteric lymph nodes (3–5 per mouse), spleen, and gall bladder. Phosphate buffered saline (PBS) was added to a total volume of 1 mL for each isolated tissue, except for intestinal contents and intestinal wall which required resuspension in a total volume of 5 mL PBS. Samples were homogenized with a TissueRuptor (Qiagen) on ice, serially diluted and plated on MacConkey agar plates containing 1% lactose and 20 μg/ml streptomycin in triplicate (both SL1344 and D23580 are resistant to streptomycin). Plates were incubated overnight at 37°C and the number of colonies enumerated the following day. The data represent an average of two independent trials and are presented as mean of either the CFU per gram of tissue or per total organ (for mesenteric lymph nodes and gall bladder). Statistical comparisons were made using the Mann-Whitney test (p < 0.05).

### Motility assays

Bacterial strains were each profiled for swimming motility on plates containing 0.3% top agar and 1.5% bottom agar containing 0.5% NaCl, 1% tryptone and 0.3% glucose. Overnight cultures of each strain were diluted 1:1000 and then spotted onto the agar using a sterile pipette tip. Plates were incubated at 37°C overnight for 8 hours. Experiments were performed in biological duplicate and technical triplicate.

### Acid stress survival assays

Bacteria were grown as described above to late log/early stationary phase, and immediately subjected to acidic conditions through the addition of a citrate buffer to lower the pH to 3.5. Cells were incubated statically at room temperature during exposure to the stress and the pH was confirmed with an electrode at the end of the assay. Samples were removed at time zero (before the addition of stress) and at various time points thereafter, diluted in phosphate buffered saline (PBS) and then plated on LB agar to determine the numbers of viable CFU. Percent survival was calculated as the number of CFU at each time point divided by the number of CFU at time zero. At least three independent trials were performed. Statistical comparisons were made using the Student’s t-test (p < 0.05).

### Biochemical analysis

Biochemical analysis of bacterial cultures was performed using the API 20E kit (bioMérieux, Durham, NC), according to the manufacturer’s instructions. The citrate test results were confirmed by using Simmons citrate media. A needle containing pure bacterial culture was tabbed twice into Simmons citrate agar slant media, and then streaked from the base of the tube up along the surface of the slant. For the catalase test, bacterial colonies of each strain were picked from plates grown overnight at 37°C on sterile polystyrene plastic petri dishes (USA Scientific). One to two drops of hydrogen peroxide was then added simultaneously to each strain and immediately imaged for bubble formation as evidence of catalase activity.

## Results

### Virulence of D23580

To assess the lethality of D23580 in mice, we performed p.o. inoculations of eight-week-old female BALB/c mice with a series of doses ranging from 10^9^ to 10^2^ CFU per dose, with five mice per dose. Results shown in Fig 1 correspond to representative data from three independent virulence assays. The LD_50_ following p.o. infection was 4.75 x 10^5^ CFU. This LD_50_ is over 4 times lower than what has been previously reported for SL1344 in BALB/c mice using bacteria cultured to the same phase of growth and an oral route of infection (2 x 10^5^–5 x 10^5^ CFU) [32,33].

### D23580 is recovered from the spleen and gall bladder of BALB/c mice at higher numbers than SL1344

**Fig 2:**
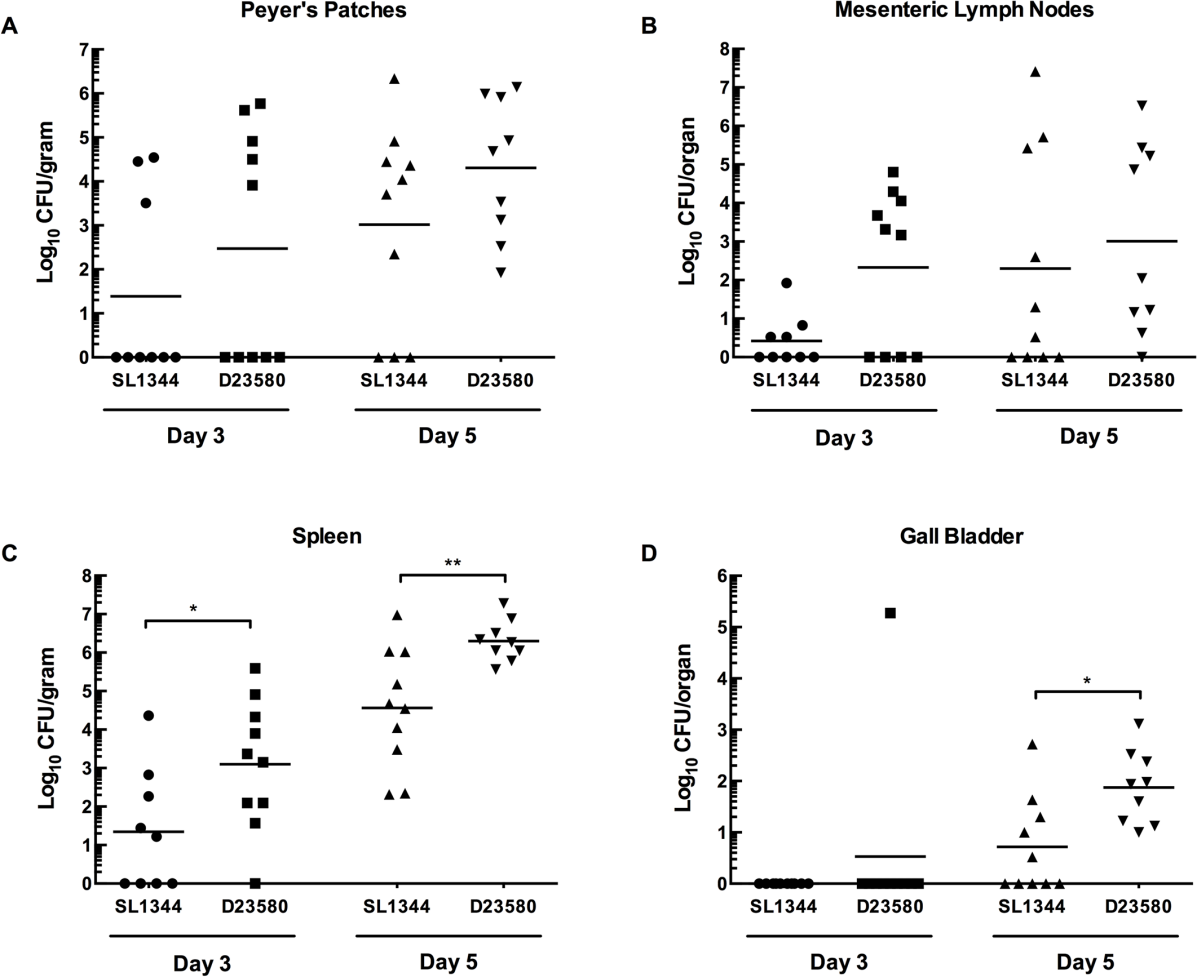
Tissue distribution of D23580 and SL1344 in mice following peroral infection. D23580 or SL1344 cultured to late log phase was administered perorally to 8-week-old female BALB/c mice at 10^8^ CFU per dose. Peyer’s patches (A), mesenteric lymph nodes (B), spleen (C) and gall bladder (D) were excised at 3 and 5 days after peroral infection. Five mice were euthanized at each time point per strain per experiment. The data represent an average of two trials and are presented as the mean of either the CFU per gram of tissue or per total organ (for mesenteric lymph nodes and gall bladder). The horizontal bar indicates geometric means and statistical comparisons were made using the Mann-Whitney test (** indicates p < 0.01; * indicates p < 0.05).

To assess the pattern of systemic spread of D23580 in mice following p.o. infection relative to the well-characterized *S*. Typhimurium strain SL1344, we infected groups of eight-week-old female BALB/c mice with either D23580 or SL1344 at 5 x 10^8^ CFU per dose and determined the CFU of the two strains in several different tissues. Quantitation of viable bacteria in tissues and organs at days 1, 3 and 5 post-infection was performed for the following: intestinal contents, Peyer’s patches, intestinal wall (small and large intestine excluding Peyer’s patches), mesenteric lymph nodes, spleen, and gall bladder. Fig 2 shows the results from 3 and 5 days post-infection, as no differences were observed one day post-infection (S2 Fig). At Day 3, no statistical differences were observed in the colonization of the Peyer’s patches of D23580 compared to SL1344 (Fig 2A; p = 0.2396), although a slight upward trend could be observed for D23580. Similarly, in the mesenteric lymph nodes an increased trend could be observed for D23580, but the differences were not statistically significant (Fig 2B; 81.3-fold, p = 0.1056). However, D23580 exhibited an enhanced ability to colonize the spleen relative to SL1344 (Fig 2C; 56.8-fold, p <0.05). No significant differences were observed for the intestinal contents or intestinal wall (excluding Peyer’s patches, S2 Fig), or for the gall bladder on day 3 (Fig 2D).

By Day 5, D23580 was still present at significantly higher levels in the spleen (Fig 2C; 54.9-fold, p < 0.01) and had spread to the gall bladder at higher counts relative to SL1344 (Fig 2D; 14.4-fold, p < 0.05), which is a hallmark of Typhi infections. The enhanced ability for D23580 to reach the deeper tissues like the spleen and gall bladder as compared to a strain belonging to the ST19 pathovar (SL1344) is consistent with previous findings by Parsons et al. that found the ST19 isolates 4/74 and F98 were slower to invade into the spleen and liver of chickens than D23580 [18].

### Acid stress resistance

**Fig 3:**
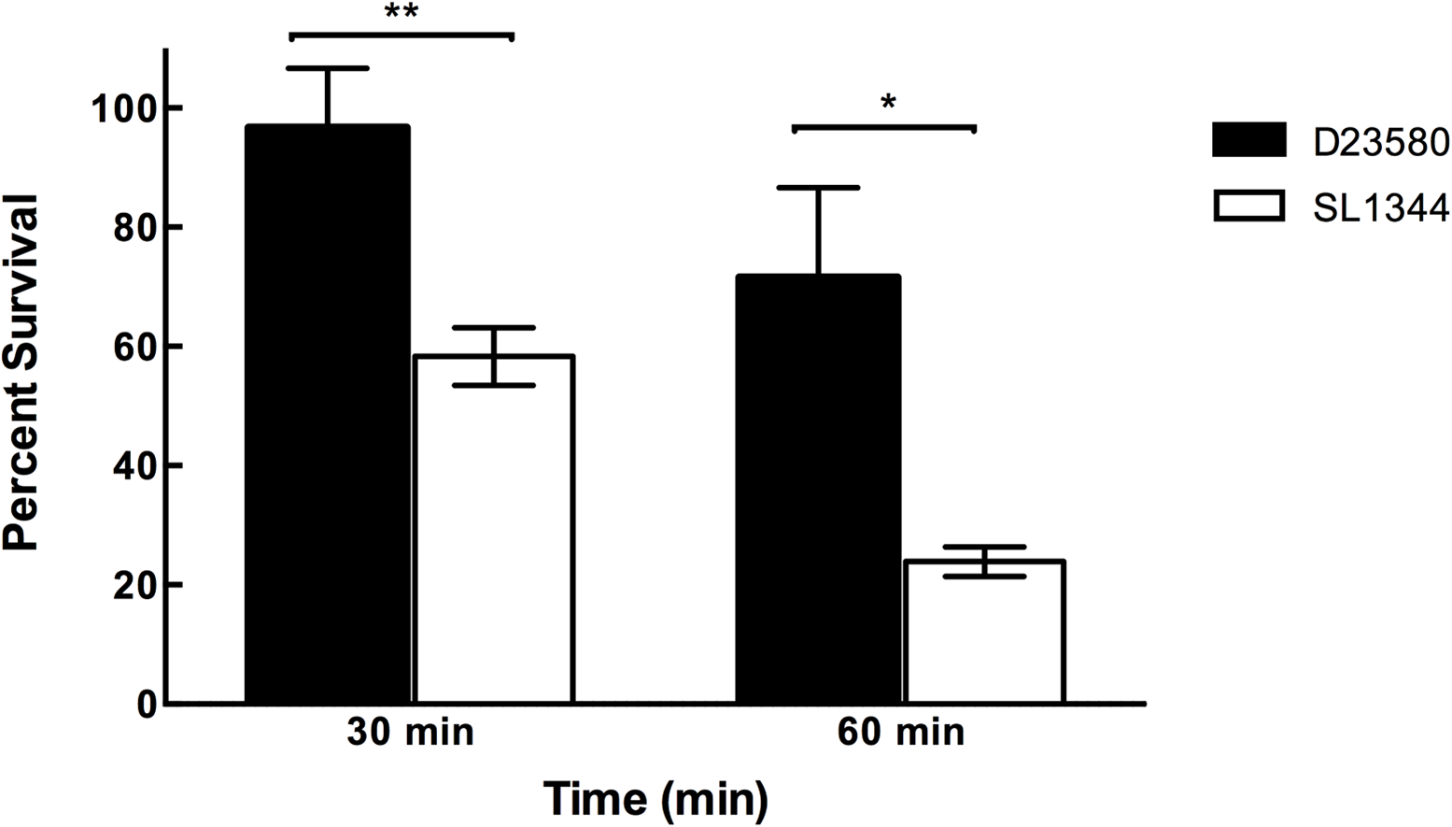
Survival of D23580 and SL1344 at pH 3.5. D23580 (black bars) and SL1344 (white bars) were cultured to late log phase, and immediately subjected to acidic conditions through the addition of a citrate buffer to lower the pH to 3.5. Samples were removed at time zero (before the addition of stress) and at various time points thereafter, diluted in phosphate buffered saline (PBS) and then plated on LB agar to determine the numbers of viable CFU. Percent survival was calculated as the number of CFU at each time point divided by the number of CFU at time zero. At least three independent trials were performed. Statistical comparisons were made using the Student’s t-test (** indicates p < 0.01; * indicates p < 0.05).

We considered that D23580 might be recovered in higher numbers from the gallbladder, spleen and MLNs as compared to SL1344 due to increased resistance to environmental stresses normally encountered in these tissues. Thus, to identify potential phenotypic traits of D23580 that may confer a selective advantage for its enhanced dissemination into these tissues as compared to SL1344, we profiled the ability of these two strains to resist low pH, a physiologically relevant stressor normally encountered by *Salmonella* both during transit through the stomach and during intracellular lifestyle within the host [34–36]. As shown in Fig 3, D23580 displayed enhanced resistance to pH 3.5 than SL1344 for all time points tested (p < 0.05).

### Motility

**Fig 4:**
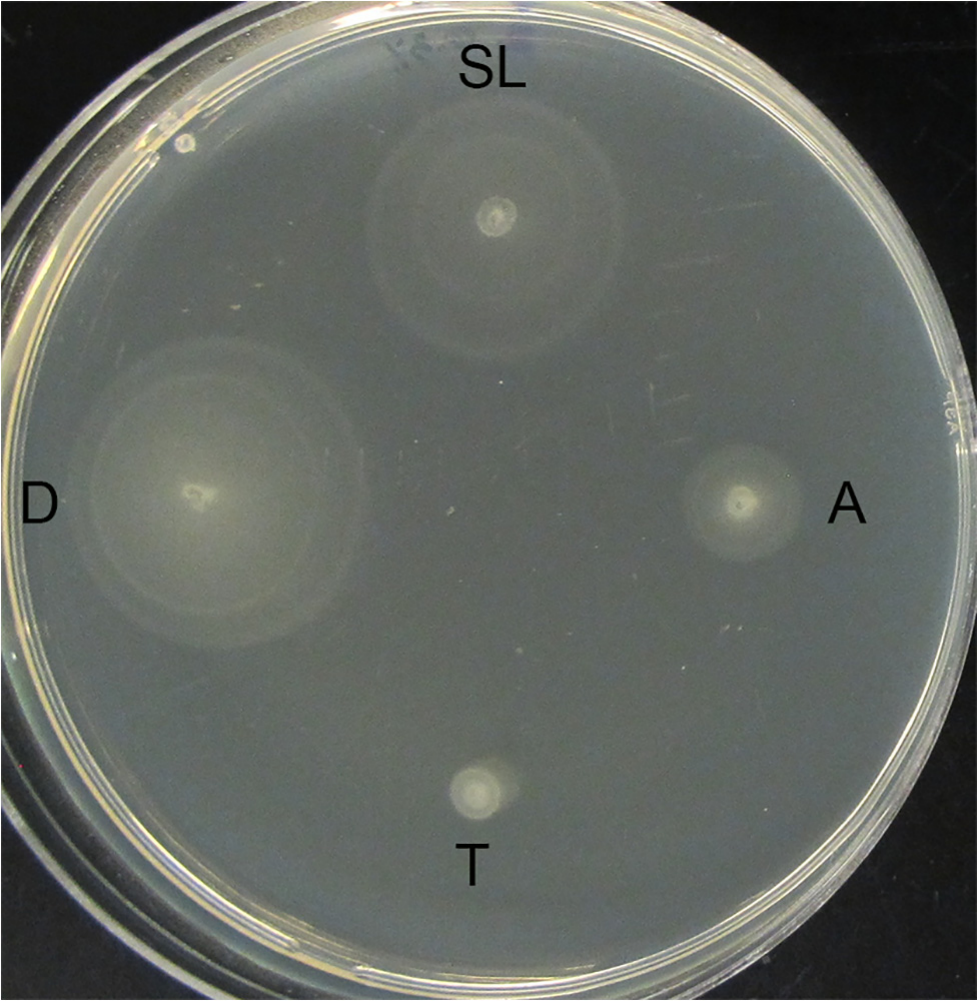
Swimming motility of ST313 strains relative to classic NTS and typhoidal strains. *Salmonella* strains D23580, SL1344, A130, and Ty2 (identified as D, SL, A, and T respectively in the figure) were each profiled for swimming motility on agar plates containing 0.3% agar, 0.5% NaCl, 1% tryptone. Overnight cultures of each strain were diluted 1:1000 and then spotted onto the agar using a sterile pipette tip. Plates were incubated at 37°C overnight for 8 hours. Experiments were performed in biological duplicate and technical triplicate.

The swimming motility of D23580 was profiled and compared to SL1344. For a broader comparison, we also included ST313 strain A130 and typhoidal strain Ty2. A130 is a chloramphenicol sensitive ST313 isolate recovered in 1997, prior to the emergence of the full MDR phenotype found in D23580 [1]. As shown in Fig 4, D23580 exhibited the highest swimming motility of all strains profiled. This heightened motility was not conserved across all ST313 isolates, as A130 appeared to be much less motile.

### Biochemical profiling

The flexible metabolic capabilities that are characteristic of enteric pathogens like *Salmonella* may confer a selective advantage during colonization of host tissues [37]. During their natural life cycle, salmonellae adapt to a wide variety of environmental niches both inside and outside of the host that vary in nutrient availability. Understanding the biochemical features that distinguish D23580 from other NTS strains may provide insight into selective pressures influencing its ability to colonize and spread within the infected host. Table 2 shows the results of the biochemical assessment. The data obtained for D23580 and SL1344 showed identical results for the ornithine decarboxylase, hydrogen sulfide production, and rhamnose and arabinose fermentation/ oxidation tests.

**Table 2:** Biochemical characterization of ST313 strains.

				Amino Acid Decarboxylation										Carbohydrate fermentation								
Test		CAT	ONPG	ADH	LDC	ODC	CIT	H_2_S	URE	TDA	IND	VP	GEL	GLU	MAN	INO	SOR	RHA	SAC	MEL	AMY	ARA
iNTS *S*. Typhimurium	D23580	+^w^	–	+	+	+	+	+	–	–	–	+^w^	–	+	+	**+**	+	+	–	**–**	–	+
ST313	A130	+^w^	–	+	+	+	+	+	–	–	–	+^w^	–	+	+	**+**	+	+	–	**+**	–	+
NTS *S*. Typhimurium	SL1344	+	–	+	+	+	–	+	–	–	–	–	–	+	+	–	+	+	–	+	–	+
S. Typhi	Ty2	–	–	+	+	–	–	+^w^	–	–	–	–	–	+	+	–	+	–	–	+	–	–

*Abbreviations*: CAT: catalase; ONPG: ortho-Nitrophenyl-β-galactoside; ADH: arginine dihydrolase; LDC: lysine decarboxylase; ODC: ornithinine decarboxylase; CIT: citrate utilization; H2S: hydogen sulfide production; URE: urease—Urea hydrolysis; TDA: tryptophan deaminase; IND: indole production- tryptophanase; VP: Voges-Proskauer—acetoin production; GEL: gelatinase; GLU: glucose fermentation / oxidation; MAN: mannitol fermentation / oxidation; INO: inositol fermentation / oxidation; SOR: sorbitol fermentation / oxidation; RHA: rhamnose fermentation / oxidation; SAC: saccharose fermentation / oxidation; MEL: melibiose fermentation / oxidation; AMY: amygdalin fermentation/oxidation; ARA: arabinose fermentation/ oxidation. The ^w^ indicates weak positive reaction.

There were striking differences that distinguished D23580 and A130 from other *Salmonella* strains tested. One difference was the ability of both ST313 strains to ferment inositol, while SL1344 and Ty2 were unable to use this carbohydrate as a sole carbon source. Inositol is produced naturally in the human body, and is found at high levels in the human brain [38], and is also found in certain foods like beans, rice and cereals as well as in soil. While certain *Salmonella* strains have the capability to utilize inositol as a carbon source, it is not ubiquitous [39]. Along these same lines, we also found that D23580 was unable to ferment melibiose, a sugar most commonly found in plants, especially legumes. In contrast, all other *Salmonella* strains, including A130, were still melibiose positive. Interestingly, although approximately 95% of *Salmonella* species are melibiose fermenters, it has been previously reported that a loss in the ability to utilize the sugar strongly correlated with clinical isolates that were associated with a *Salmonella* Enteriditis outbreak [40]. The Voges-Proskauer (VP) reaction, which is typically negative for *Salmonella* spp., was found to be weakly positive for D23580 and A130. This result indicates that these strains are capable of fermenting sugars to pyruvate via the butylene glycol pathway, which produces neutral end products, including acetoin and 2,3-butanediol. This is in contrast to other *Salmonella* pathovars, which typically produce acidic end products and as such, SL1344 and Ty2 tested negative in the VP reaction. Both D23580 and A130 presented positive reaction for the citrate test, indicating the ability for these strains to utilize citrate as the sole carbon source, while all other strains tested were negative. These results were confirmed utilizing Simmons’ citrate medium. Catalase tests revealed that while SL1344 displayed a strong positive reaction to hydrogen peroxide, both D23580 and A130 showed an extremely weak reaction, which was confirmed by performing a heavier bacterial inoculation as well (S3 Fig). This phenotype was similar to what was observed for Typhi strain Ty2, which is a naturally occurring *rpoS* mutant and thus impaired in its ability to produce catalase and resist killing by hydrogen peroxide [41,42].

## Discussion

*Salmonella* remains one of the best-characterized microbial pathogens; however we still have limited knowledge regarding the distinct pathogenesis mechanisms associated with human infections, including the invasive, MDR ST313 pathovar that has been responsible for an outbreak of iNTS infections in sub-Saharan Africa. Although genomic analysis of multiple ST313 strains, including D23580, initially indicated the possibility that this pathovar may be evolving more towards a more host-restricted phenotype like that of *S*. Typhi due to the presence of multiple gene deletions and inactivations [1], a subsequent study confirmed that D23580 and a different ST313 strain, Q456, were not host-restricted and caused an invasive disease in chickens [18]. However, no one has yet assessed the potential lethality of any ST313 strain in a non-human model.

In this study, we demonstrate that D23580 indeed causes a lethal disease in eight-week-old female BALB/c mice infected via the peroral route, with a median lethal dose of 4.75 x 10^5^ CFU. This LD_50_ value is over 4 times lower than what was previously reported for the well-characterized *S*. Typhimurium ST19 strain SL1344 [32,33]. This finding is especially intriguing, given that D23580 spread more rapidly than SL1344 into deeper tissues of the mice, including the spleen and gall bladder; a finding which is in line with previous reports demonstrating that D23580 spread faster to the spleen than ST19 strains F98, and 4/74 in chickens [18]. In addition, a study that was recently published while this manuscript was under review reported the tissue distribution of several ST313 strains in the liver, bone marrow and gall bladder of C57BL/6 mice and confirmed that the ST313 strains were indeed able to colonize these systemic sites [43]. However, the authors did not observe a statistical difference in colonization of these tissues between the ST313 isolates tested and SL1344. Differences in a number of experimental parameters could explain these seemingly divergent results, including differences in 1) the ST313 strain used to test colonization of these tissues—D23580 (this work) versus other ST313 isolates [43]; 2) mouse strains used—BALB/c (this work) versus C57BL/6 [43]; 3) route of infection—peroral (this work) versus oral gavage [43]; and 4) bacterial growth conditions.

In this study, we did not observe any statistical difference between D23580 and SL1344 in the initial colonization of the Peyer’s patches or intestinal walls (devoid of Peyer’s patches), indicating that the inherent differences between the dissemination of D23580 and SL1344 in the mouse model of infection is most likely not due to differences in the initial adherence/invasion of the pathogens to the intestinal epithelium. It is likely that the differences observed in the systemic colonization of D23580 within the mouse are multifactorial, including a combination of differences in stress resistance, survival and replication. A recent study by Ramachandran et al [26] found that D23580 survived better than SL1344 in macrophages. This trend was similar across multiple ST313 and ST19 strains profiled. Moreover, it was also found that macrophages infected with either ST313 strain D65 or ST19 strain I77 led to an induction of more proinflammatory cytokines and increased apoptosis in macrophages infected with I77 relative to those infected with D65. These factors may account for the dissemination differences between D23580 and SL1344.

During the course of infection, *Salmonella* encounters a variety of potentially lethal stressors that can alter its pathogenesis, replication and survival. The ability of the pathogen to resist these environmental insults can have a profound impact on the duration and severity of the infection. In this study, we profiled the ability of D23580 to survive exposure to low pH and found that D23580 displayed an enhanced resistance to acid stress relative to SL1344 *in vitro*. *Salmonella* encounters harsh acidic environments in the host, during its transit through the stomach as well as within the macrophage phagolysosome [35]. There is previous evidence suggesting a correlation between the acid tolerance response in *S*. Typhimurium and virulence [34]. As mentioned, no reservoir has yet been identified for the ST313 pathovar, and it has been suggested that the mode of transmission may be person-to-person rather than via the food-borne route [5,6]. Certain pathogens like *Shigella*, which are predominantly transmitted person-to-person, tend to possess a high resistance to acid killing in order to survive low gastric pH and other acidic environments *in vivo* [44–46]. It is possible that the increased acid stress resistance of D23580 may be one pathogenesis-related factor that could help to facilitate person-to-person transmission. Future investigations into the role of acid resistance in the pathogenesis of D23580 and other ST313 isolates may provide additional insight in this regard.

D23580 was also found to exhibit a greater swimming motility than all strains profiled in this study. The importance of motility for the virulence of *S*. *enterica* appears to depend on a variety of factors, including the type of host as well as the local microenvironment during infection [47–51]. In mouse models of systemic infection with *S*. Typhimurium, while motility appears to regulate some aspects of pathogenesis, it does not appear to be important for virulence [47,51,52]. However, in streptomycin-pretreated mice (which serve as a model of colitis) motility was shown to play a role in colonization and in the induction of colitis [53]. Similarly, in 1-day old chicks it was demonstrated that motility was important for both the virulence of *S*. Typhimurium as well as its persistence in the liver and spleen [54]. In a calf model of enterocolitis, flagella were found to be required for maximum fluid secretion and for the influx of polymorphonuclear leukocytes during infection [51]. *In vitro* infection assays have also been used to profile the impact of motility on the ability for *S*. Typhimurium to attach and invade into cells [48,50,51,55–58]. While many of these infection studies using flat 2-D monolayer cultures have indicated that the motility is important for the colonization of the intestinal epithelium, a recent study using our 3-D organotypic model of human intestinal epithelium revealed that an *flhDC* flagellar mutant was still able to actively invade at much greater levels than in 2-D monolayers without the need for centrifugation during the adherence step, although still to a lesser extent than wild type [59].

Of particular relevance to ST313 strains, it was previously found that the physiological origin of clinical isolates might also impact motility in that *S*. Typhi clinical isolates of blood-borne origin displayed a significantly higher swimming motility than stool-borne strains [60]. In addition, a previous study reported that while flagella-mediated motility was not required for invasion, it does lead to enhanced invasion although it was not absolutely required for invasion to occur [50]. Thus it is possible that the greater motility observed in this study for D23580 may be associated with the enhanced tissue distribution in systemic mice organs as compared to SL1344. D23580, which is blood-borne in origin, displayed enhanced motility in our present study relative to other strains profiled. However, in our present study we observed a sizeable difference in the motility between the blood-borne ST313 clinical isolates, D23580 and A130, in that A130 was much less motile. A recent comparative study of several ST313 strains (S12, Q55, D65, and S11) with ST19 strains (I77, S52, I41, and I89) found that the ST313 strains were significantly less motile and produced less flagellin than the ST19 strains [26]. Additional studies are needed to better understand the potential role of this enhanced response on the infection properties of D23580.

In an effort to identify possible metabolic characteristics of D23580 associated with its enhanced ability to reach deep tissues in mice following p.o. infection, we performed a series of biochemical analyses to identify differences between this ST313 strain and classic *Salmonella* pathovars. Key differences were observed for D23580 for three biochemical tests, including melibiose and inositol utilization and the Voges-Proskauer test. In particular, it is intriguing that both ST313 strains were able to use inositol, since 1) increased inositol levels are found early in the course of HIV-related brain disease [61] and ST313 infections in adults are strongly associated with HIV infected individuals [8,62,63], and 2) inositol metabolism is important for the intraerythrocytic development of the malarial parasite *Plasmodium falciparum* [64] and infants with malaria are at high risk for ST313 infections [1,65,66]. In addition to the differences described above, D23580 was also the only strain unable to use melibiose–a distinct characteristic that has been associated with another clinical outbreak [40], and may be useful for discriminating isolates during biotyping. Relevant to these findings, Okoro *et al*. recently reported single nucleotide polymorphisms and pseudogenes associated with metabolism in ST313 isolates and experimentally showed using phenotype microarrays that these isolates differed from ST19 strains (including reference strain ST4/74, which is the parental strain of SL1344) in their utilization of select carbon sources, including L-tartaric acid, tricarballyic acid and meso-tartaric acid [43]. In addition, it was observed that ST313 strains more readily used melibionic acid (a derivative of melibiose), than the ST19 strains tested [43]. Collectively, our observations may provide clues for future studies into the metabolic adaptation and pathogenic mechanisms of ST313 strains.

## Supporting Information

S1 Fig Growth curves of D23580 and SL1344. Bacterial cultures were initiated in LB with aeration (180 rpm) overnight for 15 hours at 37°C. The following day, overnight cultures were inoculated into 5 mL sterile LB at a 1:200 dilution and subsequently grown at 37°C with aeration. Cultures were monitored by plating on LB agar for viable colony-forming units (CFU) and measuring the corresponding optical density at 600 nm (OD_600_).
(TIFF)

S2 Fig Tissue distribution of D23580 and SL1344 in mice following peroral infection. D23580 or SL1344 cultured to late log phase was administered perorally to 8-week-old female BALB/c mice at 10^8^ CFU per dose (5 mice total). The bacterial load in Peyer’s patches and mesenteric lymph nodes on Day 1 following infection (A) as well as within the intestinal contents and the intestinal wall (excluding Peyer’s patches) on Days 1, 3, and 5 (B, C) are shown. The data are presented as the mean of either the CFU per gram of tissue or per total organ (mesenteric lymph nodes). The horizontal bar indicates geometric means. The data shown represent a single experimental trial using five mice. Since no statistical differences were observed on Day 1 for any tissue or for Days 3 and 5 for the intestinal wall or intestinal contents, these experiments were not replicated.
(TIFF)

S3 Fig ST313 strains display a weakly positive catalase reaction. Bacterial colonies of each strain (D23580, SL1344, A130 and Ty2) were picked onto sterile polystyrene plastic petri dishes from plates grown overnight at 37°C. One to two drops of hydrogen peroxide was then added simultaneously to each strain and immediately imaged for bubble formation.
(JPG)
