# Supplementary figures and images for: Characterization of the Invasive, Multidrug Resistant Non-typhoidal Salmonella Strain D23580 in a Murine Model of Infection
Source: PLoS Negl Trop Dis. 2015 Jun 19;9(6):e0003839. doi: 10.1371/journal.pntd.0003839 (PMC4474555; doi:10.1371/journal.pntd.0003839)

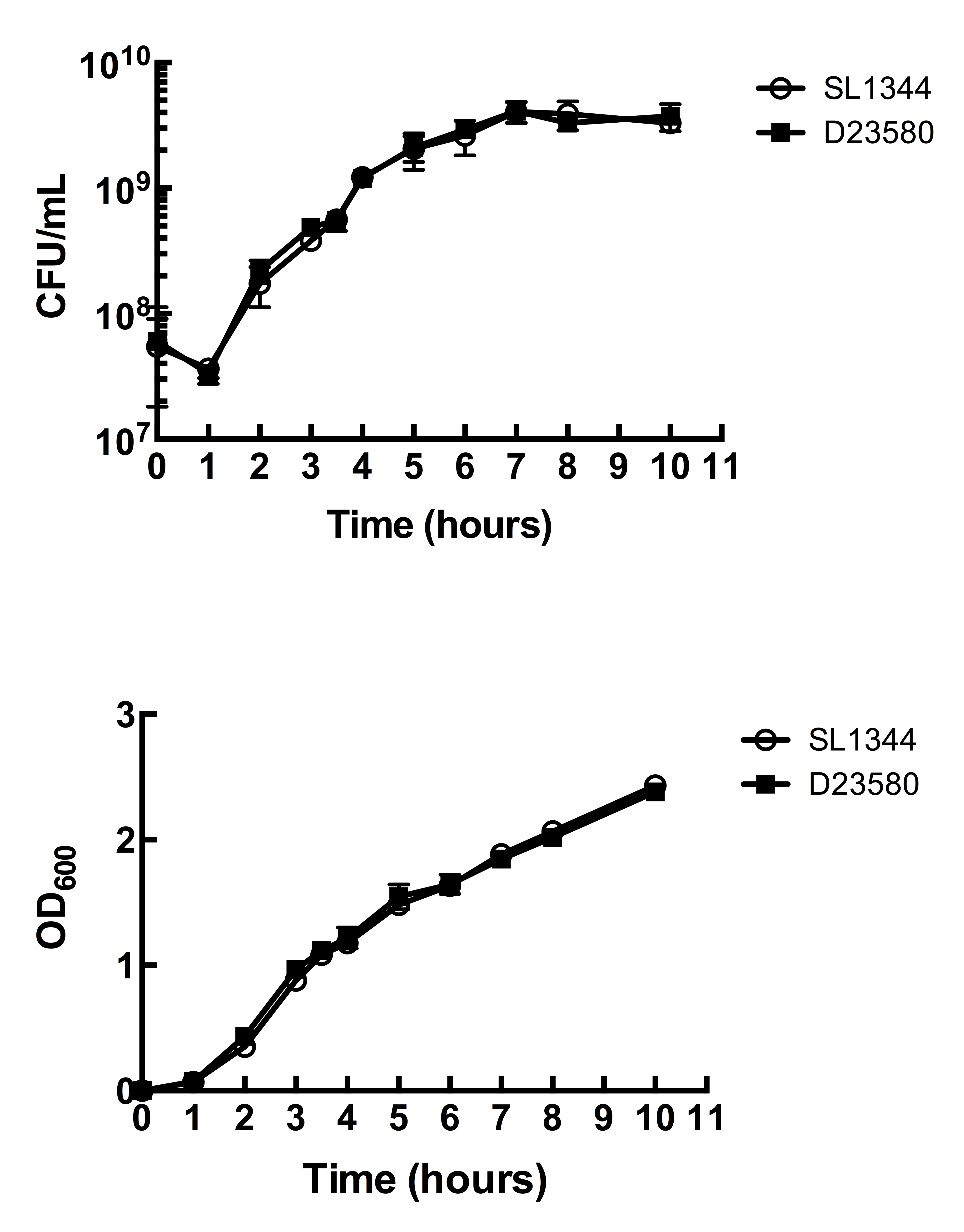

Supplement: S1 Fig — Bacterial cultures were initiated in LB with aeration (180 rpm) overnight for 15 hours at 37°C. The following day, overnight cultures were inoculated into 5 mL sterile LB at a 1:200 dilution and subsequently grown at 37°C with aeration. Cultures were monitored by plating on LB agar for viable colony-forming units (CFU) and measuring the corresponding optical density at 600 nm (OD600). (TIFF) [file pntd.0003839.s001.tiff]

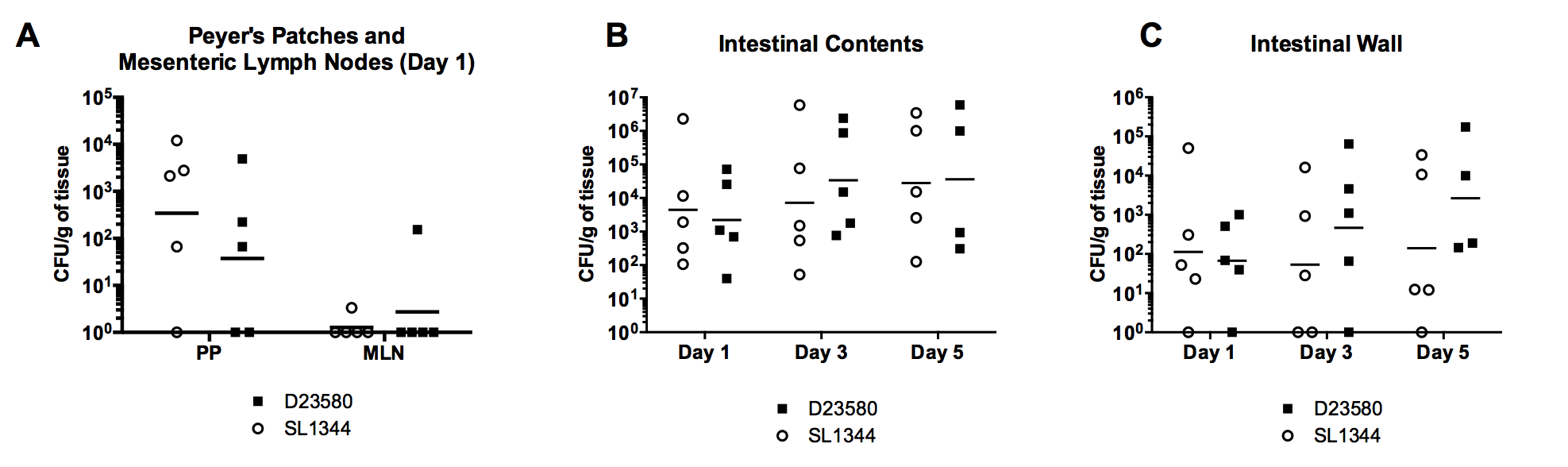

Supplement: S2 Fig — D23580 or SL1344 cultured to late log phase was administered perorally to 8-week-old female BALB/c mice at 108 CFU per dose (5 mice total). The bacterial load in Peyer’s patches and mesenteric lymph nodes on Day 1 following infection (A) as well as within the intestinal contents and the intestinal wall (excluding Peyer’s patches) on Days 1, 3, and 5 (B, C) are shown. The data are presented as the mean of either the CFU per gram of tissue or per total organ (mesenteric lymph nodes). The horizontal bar indicates geometric means. The data shown represent a single experimental trial using five mice. Since no statistical differences were observed on Day 1 for any tissue or for Days 3 and 5 for the intestinal wall or intestinal contents, these experiments were not replicated. (TIFF) [file pntd.0003839.s002.tiff]

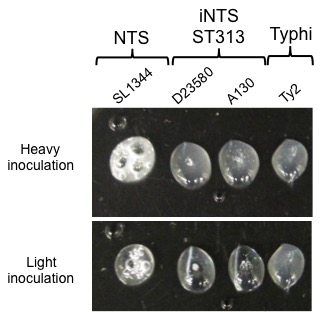

Supplement: S3 Fig — Bacterial colonies of each strain (D23580, SL1344, A130 and Ty2) were picked onto sterile polystyrene plastic petri dishes from plates grown overnight at 37°C. One to two drops of hydrogen peroxide was then added simultaneously to each strain and immediately imaged for bubble formation. (JPG) [file pntd.0003839.s003.jpg]
